# The complete mitochondrial genome of the chiltepin pepper (*Capsicum annuum* var. *glabriusculum*), the wild progenitor of *Capsicum annuum* L.

**DOI:** 10.1080/23802359.2020.1714496

**Published:** 2020-01-16

**Authors:** Mahmoud Magdy, Bo Ouyang

**Affiliations:** a Key Laboratory of Horticultural Plant Biology (Ministry of Education), Huazhong Agricultural University, Wuhan, China;; b Genetics Department, Faculty of Agriculture, Ain Shams University, Cairo, Egypt

**Keywords:** Chiltepin pepper, plant mitogenome, NGS, pepper genomics

## Abstract

The complete mitochondrial genome of chiltepin pepper (*Capsicum annuum* var *glabriusculum*) was sequenced. The mitogenome of the American bird pepper was 505,190 bp, with 44.4% of GC content. A total of 218 genes were fully annotated, including 190 CDS (31 known genes and 158 open reading frames), three rRNA, and 25 tRNA genes. The gene synteny and number were equal to those of *C. annuum* var *annuum*, except for the partial annotation of ATP subunit 6 and the absence of ORF172 and ORF104b. The complete mt genome sequence was deposited to the GenBank (NCBI, Accession number: MN196478).

Peppers are increasingly essential vegetables worldwide and frequently used for culinary and medicinal purposes. Chiltepin pepper (*Capsicum annuum* L. var. *glabriusculum*) is a member of the Solanaceae family. It is considered the ancestor of the cultivated chili and bell pepper, *Capsicum annuum* var. *annuum* (Pickersgill 1988), the most economically important domesticated *Capsicum* species. Its genome sequence was published (Qin et al. 2014), the plastome was reported (Raveendar et al. 2015), and resequenced for comparative plastomics (e.g. Magdy et al. 2019); however, the mitochondrial genome has yet to be published. This reported mitogenome sequence will provide a valuable extranuclear-genetic background of the American bird pepper, the wild progenitor of *C. annuum* species for further genomic-based analysis.

In this study, we reported and characterized the complete mitochondrial genome of the wild progenitor, American bird pepper (*C. annuum* var. *glabriusculum*, CAG). The whole-genome sequence reads were generated through DNA extraction of a wild CAG accession retrieved from USDA-ARS that was originally collected from Mexico (USDA-ARS: PI 593546, https://npgsweb.ars-grin.gov/gringlobal/accessiondetail.aspx?id=1515344) and paired-end whole-genome shotgun resequencing using Illumina HiSeq 2000 (Novogene, China) with ∼300 bp insert size at 11× sequence depth. Clean pair-end reads were filtered and mapped to the *C. annuum* published mitogenome (NC_024624; Jo et al. 2014) with five iterations times to generate a preliminary mitogenome. Followed by a remapping to the preliminary mitogenome with extra ten iterations to finally construct the final CAG mitogenome using Geneious Prime (Kearse et al. 2012). The CAG mitogenome was annotated as a circular molecule using GeSeq – Annotation of Organellar Genomes (Tillich et al. 2017) based on the *C. annuum* mitogenome (NC_024624), whereas tRNAscan-SE V239 was used to find and annotate tRNA genes. All the annotated CDS were verified by translation using Geneious Prime. The mitogenomes of relative species belong to the family Solanaceae available from NCBI database were downloaded and aligned along with the CAG mitogenome using Mauve aligner (Darling et al. 2004). The phylogenetic analysis was performed using Fasttree 2.1.5 (Price et al. 2010).

The total mapped clean pair-end reads were 6,725,324 reads. The complete mitogenome of the American bird pepper was 505,190 bp, which was deposited to GenBank (No. MN196478). The GC content of the mitogenome was 44.4%. A total of 218 genes were fully annotated, with 190 CDS (31 genes and 158 open reading frames), three rRNA, and 25 tRNA genes. Among these genes, five, two, and four of them contained one, three, or four introns, respectively. The genes with known functions were ATP synthase subunits (*atp* 1 and 9), apocytochrome b (*cob*), cytochrome c maturation protein (*ccm* B, C, Fc, and FN), cytochrome oxidase subunits (*cox* 1, 2, and 3), maturase-related protein (*mat*-R), NADH dehydrogenase subunits (*ndh* 1, 2, 3, 4, 4L, 5, 6, 7, and 9), ribosomal protein large subunits (*rpl* 2, 5, and 10), ribosomal protein small subunits (*rps* 3, 4, 10, 12, 13, and 19) and succinate dehydrogenase subunits (*sdh* 3 and 4). The CAG mitogenome contains 25 different tRNA genes, three in which were duplicated (*trn*C, *tnr*L, and *trn*P), one was triplicated (*trn*S), and one was tetra-plicated (*trn*M) along the mitogenome. Compared to *C. annuum* reference, the ATP subunit 6 was partially annotated, whereas ORF172 and ORF104b were not annotated. The CAG was clustered along with *C. annuum* var *annuum*, forming the *Capsicum* genus clade, sister to the *Solanum* genus clade and adjacent to the *Nicotiana* genus clade. All of them were rooted with *Hyoscyamus niger*, and all clades were highly supported (bootstrap value = 1.00; Figure 1).

**Figure 1.**
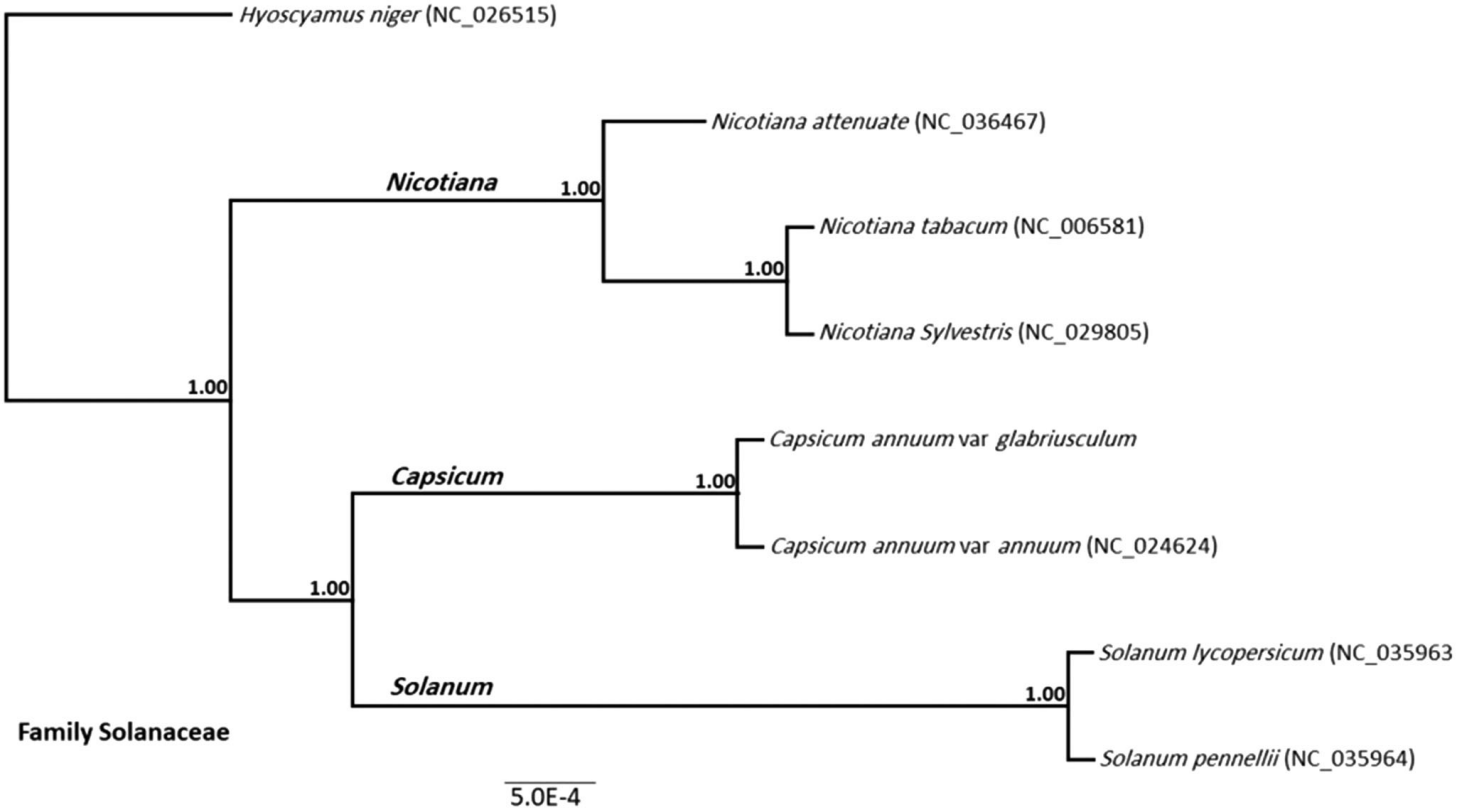
Phylo-mitogenomic analysis of the Solanaceae mitogenomes available in NCBI database. Three genera (*Nicotiana*, *Capsicum*, and *Solanum*) were defined by a separate high-supported cluster (Bootstrap support = 1.00). The studied *Capsicum annuum* var. *glabriusculum* was included in the capsicum clade, along with the published complete mitogenome of the commercially important pepper (*Capsicum annuum* var. *annuum*; NC_024624).
